# Exploring mutation specific beta blocker pharmacology of the pathogenic late sodium channel current from patient-specific pluripotent stem cell myocytes derived from long QT syndrome mutation carriers

**DOI:** 10.1080/19336950.2022.2106025

**Published:** 2022-08-10

**Authors:** Thomas W. Comollo, Xinle Zou, Chuangeng Zhang, Divya Kesters, Thomas Hof, Kevin J. Sampson, Robert S. Kass

**Affiliations:** Department of Molecular Pharmacology and Therapeutics, Columbia University Irving Medical Center, Vagelos College of Physicians and Surgeons, Columbia, NY, USA

**Keywords:** Long QT syndrome, propranolol, induced pluripotent stems

## Abstract

The congenital long QT syndrome (LQTS), one of the most common cardiac channelopathies, is characterized by delayed ventricular repolarization underlying prolongation of the QT interval of the surface electrocardiogram. LQTS is caused by mutations in genes coding for cardiac ion channels or ion channel-associated proteins. The major therapeutic approach to LQTS management is beta blocker therapy which has been shown to be effective in treatment of LQTS variants caused by mutations in K^+^ channels. However, this approach has been questioned in the treatment of patients identified as LQTS variant 3(LQT3) patients who carry mutations in *SCN5A*, the gene coding for the principal cardiac Na^+^ channel. LQT3 mutations are gain of function mutations that disrupt spontaneous Na^+^ channel inactivation and promote persistent or late Na^+^ channel current (I_NaL_) that delays repolarization and underlies QT prolongation. Clinical investigation of patients with the two most common LQT3 mutations, the ΔKPQ and the E1784K mutations, found beta blocker treatment a useful therapeutic approach for managing arrhythmias in this patient population. However, there is little experimental data that reveals the mechanisms underlying these antiarrhythmic actions. Here, we have investigated the effects of the beta blocker propranolol on I_NaL_ expressed by ΔKPQ and E1784K channels in induced pluripotent stem cells derived from patients carrying these mutations. Our results indicate that propranolol preferentially inhibits I_NaL_ expressed by these channels suggesting that the protective effects of propranolol in treating LQT3 patients is due in part to modulation of I_NaL_.

## Introduction

The congenital long QT syndrome (LQTS), first described in 1957 [1,2], is one of the most common cardiac channelopathies [3,4]. Congenital LQTS is a disorder characterized by delayed ventricular repolarization reflected in a prolongation of the QT interval of the surface electrocardiogram (EKG) that in turn is caused by mutation-induced prolongation of ventricular muscle cell action potentials [5,6]. There are now 17 different genetic subtypes of LQTS caused by mutations in genes coding for ion channels or ion channel associated proteins [6]. The variants of LQTS were named after the chronological order in which the key genes were identified. The first two genes identified coded for two key cardiac potassium channels: mutations in *KCNQ1* the α subunit of the I_KS_ channel are LQT1 mutations [7,8] and mutations in *KCNH2* the gene coding for hERG, the α subunit of I_Kr_ channels are LQT2 mutations [9,10]. All LQT1 and LQT2 mutations give rise to loss of functional activity of the coded potassium channels that in turn delay repolarization in ventricular muscle cells expressing these genes [6]. Importantly, the I_Kr_ channel has also been identified as an off-target effector site of a large number of drugs. This effect also underlies drug-induced LQTS causing pathophysiology very similar to congenital LQT2 [11,12].

Mutations in *SCN5A*, the gene coding for Na_V_1.5, the α subunit of the principal cardiac sodium channel were first reported by Keating and colleagues in 1995 [13]. A transient, or peak Na_V_1.5 channel current (I_NaP_) underlies cardiac excitation, the channels conducting I_NaP_ normally close, or inactivate, during the plateau phase of the ventricular action potential that is responsible for the QT interval of the EKG [14]. In contrast to LQT1 and LQT2 mutations, LQT3 mutations are gain of function mutations in that they disrupt channel inactivation causing an increase in Na^+^ channel activity. The hallmark functional effect of LQT3 mutations is an increase in persistent or late Na^+^ channel current (I_NaL_) that can produce a prolonged action potential plateau, and prolong the QT interval of the EKG [6]. Enhancement of I_NaL_ is often arrhythmogenic and is a drug target for LQT3 therapeutics [14,15].

LQT1 and LQT2 account for almost 85% of all genotyped LQTS patients, and LQT3 accounts for from 5% to 10% of LQTS patients [5]. Triggers for cardiac events in LQTS patients differ according to the underlying LQTS gene, and there are marked differences in events that may cause higher risk of rhythm disturbances [16]. The major therapeutic approach to management of LQTS is beta blocker therapy [3,17], and this rationale is based largely on the arrhythmia risk of LQT1 patients to stimulation of the sympathetic nervous system during exercise [16,18]. β-adrenergic actions enhanced during exercise include an increase in heart rate, an increase in L-type calcium channel current and modulation of intracellular calcium dynamics [19]. The wide use of β-blockers is due in part to this risk being modified by the positive response of LQT1 patients to β-blocker therapy which blunts this adrenergic activity [20–22] and to the fact that LQT1 is the predominant LQTS variant [5].

Because LQT3 patients are at greater risks of serious cardiac events in the setting of slow heart rates [16] it had been proposed that the use of β-blocker therapy would not be useful for treating LQT3, a concept that has been addressed in multiple studies [4,23,24]. Two of the most common LQT3 mutations are the mutation that causes the deletion of three amino acids, KPQ, in the inactivation gate of the Na_V_1.5 channel, referred to as the ΔKPQ mutation [13]; and the E1784K mutation of the Na_V_1.5 carboxy terminus [25]. Both mutations cause enhanced persistent or late Nav1.5 channel current, I_NaL_, when expressed in heterologous systems [26–29]. Schwartz and colleagues tested the antiarrhythmic activity of the beta blocker propranolol in transgenic mice expressing ΔKPQ mutant channels and found propranolol to be effective in preventing arrhythmias in this animal model [30]. Fabritz et al. suggested that these antiarrhythmic effects were likely due in part to Na^+^ channel modulation in addition to a classical anti-adrenergic effect [31]. This work was followed by a large international clinical study in which the effects of β-blocker therapy were investigated in LQT3 genotyped patients [32]. Importantly, this study included 70 patients carrying the E1784K mutation and 64 patients carrying the ΔKPQ mutation. The results of this study supported the findings of the animal studies in that β-blocker therapy was effective in preventing arrhythmia risk, particularly in females, a result that has been supported by meta-analysis [33].

The purpose of this study was to investigate the effects of the β-blocker propranolol on mutant Na^+^ channels expressed in induced pluripotent stem cell (iPSC) myocytes derived from patients carrying either the ΔKPQ or E1784K mutation or from patients who were mutation-free. The use of patient-derived iPSC myocytes allowed us to investigate the effects of propranolol not only on mutant and wild-type Na_V_1.5 channels in human cells, but to also test for possible off target effects of propranolol on I_Kr_ channels that are also expressed in these cells [34]. Our results indicate that propranolol preferentially inhibits I_NaL_ suggest that the protective effects of propranolol in treating LQT3 patients, is due to modulation of Na_V_1.5 channels along with its anti-adrenergic actions.

## Methods

### Human fibroblast reprogramming, characterization, differentiation, and culturing of iPSCs

Human fibroblasts were reprogrammed and characterized, and IPSC culture and differentiation were performed as previously described [34,35].

### IPSC-CM dissociation

Preparations containing IPSC-CMs were dissociated 25 to 60 days after differentiation. Cells were first washed with a Ca^2+^ free buffer containing 120 mM NaCl, 5.4 mM KCl, 5 mM MgSO_4_, 5 mM Na-pyruvate, 20 mM Glucose, 20 mM Taurine, and 10 mM HEPES. They were then dissociated at 37°C using 0.25% trypsin for ~8 to 28 minutes. This reaction was then quenched with 10% FBS in the before mentioned Ca^2+^ free buffer. Cells were then resuspended in DMEM supplemented with 10% FBS, 1 mM Na-pyruvate, 100 U/ml penicillin, 100 µg/ml streptomycin, and 2 mM l-glutamine. The cells were plated on 35-mm Petri dishes that had been coated with 0.1% gelatin. Single IPSC-CMs were identified based on beating status and/or morphology using a Nikon object marker and patch clamped 3 to 7 days after dissociation.

### Single-cell electrophysiology

I_NaL_ was recorded as 1 μM tetrodotoxin (TTX)-sensitive current in previously described internal and external solutions [34]. For recording I_NaL_ the external solution also contained 1 μM isradipine to block L-type Ca channels. I_NaP_ recordings used in Figure 2 and all steady state inactivation (SSI) recordings were recorded in a reduced Na^+^ external solution, containing 30 mM NaCl, 10 mM HEPES, 5 mM Glucose, 105 mM TEA-Cl, 2 mM CaCl_2_, and 1.2 mM MgCl_2_, pH was adjusted to 7.4 with TEA-OH. When studying I_NaP_ and SSI, the internal solution contained 10 mM NaCl, 2.5 mM Na_2_-ATP, 125 CsCl, 2 mM MgCl_2_, 2 mM MgCl_2_, 1 mM CaCl_2_, 10 mM EGTA, and 10 mM HEPES. pH was adjusted to pH 7.2 with CsOH. I_NaL_ and I_Na,P_ were measured as the average of the TTX-sensitive currents. I_NaL_ measurements were based on the last ~5 ms of a 100 ms pulse to −10 mV from a holding potential of −90 mV. The external solution for I_Kr_ measurement contained 132 mM NaCl, 4.8 mM KCl, 2 mM CaCl_2_, 1.2 mM MgCl_2_, 10 mM HEPES, and 5 mM Glucose with 1 μM isradipine and 30 μM chromanol 293B added prior to recording. Pipette internal solution for I_Kr_ measurements contained 110 mM KCl, 5 mM ATP-K_2_, 11 mM EGTA, 10 mM HEPES, 1 mM CaCl_2_, and 1 mM MgCl_2_. All solutions were prepared using double distilled H_2_O.

**Figure 1.**
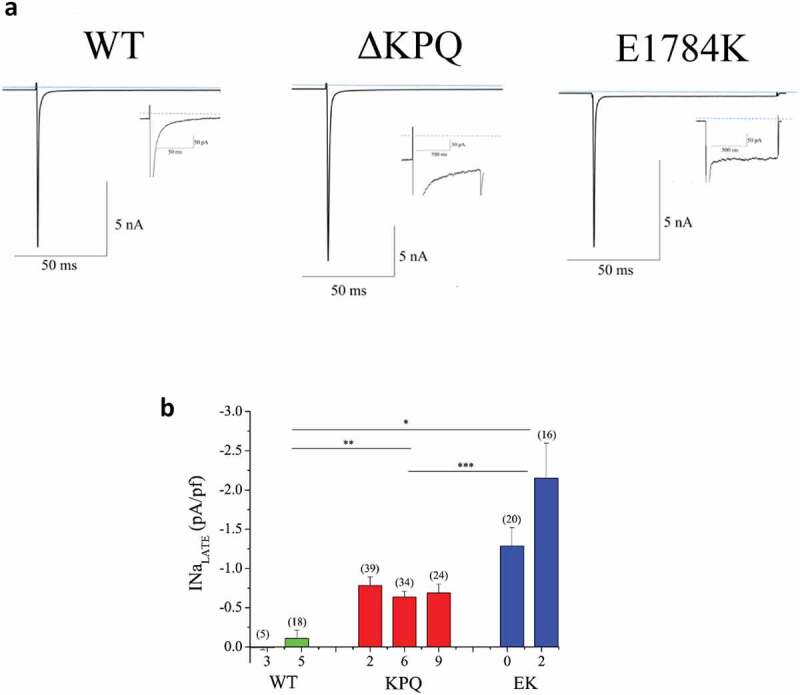
Characterization of I_NaL_ in WT and patient derived ΔKPQ and E1784K IPSC-CMs. A. Representative TTX sensitive traces of total I_Na_ and I_NaL_ (inset) from WT, ΔKPQ, and E1784K IPSC-CMs. B. I_NaL_ of each cell line quantified as percentage of I_NaP_. When two tailed unequal variance t-tests are performed on cells types (WT, ΔKPQ, and E1784K) as a whole, E1784K and ΔKPQ, had significantly more I_NaL_ than WT (p = 6.5638E-06 and 6.15E-05 respectively). E1784K also had significantly more I_NaL_ than ΔKPQ (p = 0.000235918). Currents were elicited by pulsing from a holding potential of −90 mV to −10 mV for 100 ms.

**Figure 2.**
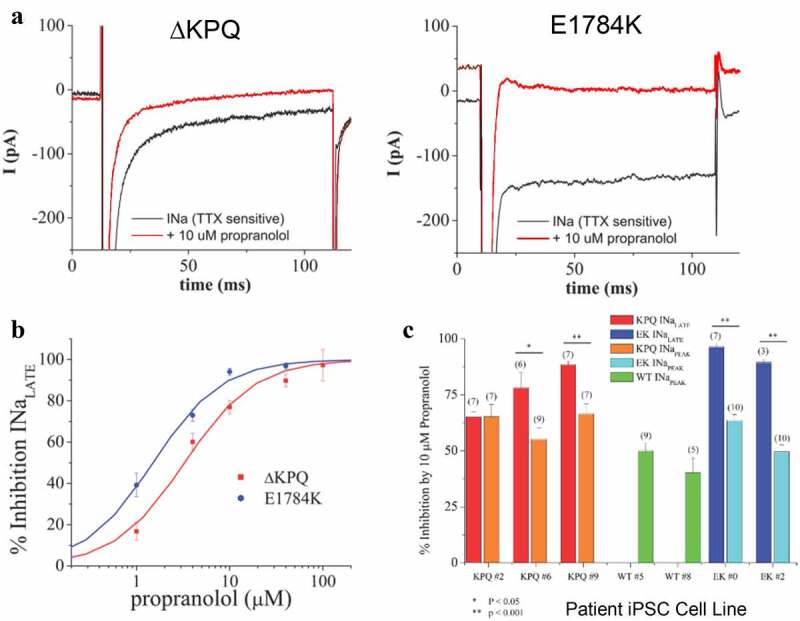
Propranolol modulation of I_NaL_ and I_NaP_ in patient derived ΔKPQ and E1784K IPSC-CMS. A. Representative high gain TTX-sensitive traces in the absence (black traces) and presence of 10 μM propranolol (red traces) reveal inhibition of I_NaL_ in ΔKPQ and E1784K IPSC-CMs. B. Concentration response curves for patient derived IPSC-CM total populations from three ΔKPQ patients (ΔKPQ2, ΔKPQ6, and ΔKPQ9) and two E1784K patients (EK0 and EK2). IC_50_ for propranolol inhibition of ΔKPQ patients’ cells combined is 3.3 μM. IC_50_ of propranolol inhibition of E1784K iPSC myocytes averaged is 1.5 μM. C. Propranolol (10 μM) inhibition of I_NaL_ and I_NaP_ for each cell line investigated. Currents were elicited using the protocol given in. Figure 1

### Statistics

Statistics were performed using in Excel 2016 Student’s t-test or Excel One Way ANOVA. When not specified, a significance p value threshold of 0.01 was used.

### Data Availability

We shall share all data used in this study.

## Results

### INaL in cells derived from mutation-free (WT) and mutation carrying (ΔKPQ or E1784K) patients

Na_V_1.5 channel activity in WT and LQT3 patient-derived IPSC-CMs harboring either the Na_V_1.5 ΔKPQ or E1784K mutation was characterized by whole cell voltage clamp. As described in Methods, cells were held at −90 mV and pulsed to −10 mV for 100 ms (Figure 1(a) (top) with a 5s inter-pulse interval. Representative traces of current recorded at low and high gain are shown in Figure 1(a). Low gain records illustrate I_NaP_ measurements. For each cell studied high gain recordings are shown as insets. Note that I_NaL_ is clearly detected for ΔKPQ and E1784K expressing cells but not for the WT cell. I_NaL_ was measured as the average current (n = 9 each cell) remaining in the last 5 ms of the pulse and was determined as TTX-sensitive current. Averaged I_NaL_ was determined in each cell line as follows. We examined two WT IPSC-CM cell lines that had minimal late Na^+^ current (0.01 ± 0.03 and −0.11 ± 0.10 pA/pf in WT3 and WT5 IPSC-CM respectively). I_NaL_ was significantly larger than WT in all mutant expressing cells studied. For the ΔKPQ cell lines, all three had significant I_NaL_(−0.78 ± 0.01, −0.64 ± 0.08, −0.91 ± 0.11 pA/pf ΔKPQ2, ΔKPQ6 and ΔKPQ9). In the E1784K mutant expressing IPSC-CMs, EK0 and EK2, I_NaL_ was −1.28 ± 0.24 and −2.15 ± 0.45 pA/pf, respectively. Figure 1(b), which illustrates the summary data for these experiments, indicates that cells expressing E1784K and ΔKPQ mutant channels had significantly more I_NaL_ than WT cells. Comparing total populations across cell lines, E1784K had significantly more I_NaL_ than the total ΔKPQ population (p = 2.4 E-04).

### Propranolol inhibits INaL expressed in IPSC-CMs

We then investigated I_NaL_ inhibition by propranolol for concentrations ranging between 1 and 100 μM. Representative traces demonstrating inhibition by 10 μM propranolol for ΔKPQ and E1784K cells are shown in Figure 2(a). Figure 2(b) summarizes average percent inhibition of I_NaL_ measured in all cell lines that we studied along with best fit concentration response curves for all cell lines for both mutants. Concentration response curves were determined using a Hill equation that provided IC_50_ values. The fitted curves revealed the following IC_50_ values for inhibition of I_NaL_: 3.36 ± 0.61 μM for cells expressing the ΔKPQ mutation, and 1.58 ± 0.04 μM for cells expressing the E1784K mutation. Inhibition of I_NaL_ ΔKPQ expressing cells was similar across all individual cell lines with the following IC_50_ values: 4.48 μM, 2.39 μM, and 3.21 μM, for ΔKPQ2, ΔKPQ6, and ΔKPQ9, respectively. In cells expressing E1784K mutant channels I_NaL_ in EK0 and EK2 was inhibited by propranolol with IC_50_ values of 1.48 μM and 1.56 μM, respectively (Please see Table 1).

**Table 1.** I_NaL_ IC_50_ values for patient-derived IPSC-CM total populations from three ΔKPQ patients (ΔKPQ2, ΔKPQ6, and ΔKPQ9) and two E1784K patients (EK0 and EK2).

Patient cell line	I_NaL_ propranolol IC_50_ (μM)
ΔKPQ2	4.48
ΔKPQ6	2.39
ΔKPQ9	3.21
EK0	1.48
EK2	1.56

### Propranolol inhibition of INaL is more potent than inhibition of INaP

We next measured the relative inhibition of I_NaL_ and I_NaP_ by propranolol applied at the high concentration of 10 μM and summarize the results for each cell line studied in Figure 2(c). For these recordings Na^+^ was reduced in the external solution (30 mM NaCl) to lower current I_NaP_ amplitude to maintain voltage control for measurement of I_NaP_ and still resolve I_NaL_. On average, we found I_NaP_ expressed in WT, ΔKPQ, and E1785K IPSC-CMs was inhibited 46.31 ± 3.46, 61.39 ± 3.16, and 56.30 ± 2.7, respectively, by propranolol. Figure 2(c) illustrates the results of these experiments summarized for each cell line studied. Inhibition of I_NaL_ vs I_NaP_ was significantly greater in each of the E1784K cells lines (EK0 p = 3.65e-07 and EK2 p = 6.60e-05) and two of the three ΔKPQ IPSC-CM cell lines (p < 0.05, p = 0.02, and 0.01). However, propranolol inhibition of I_NaL_ vs I_NaP_ was not significantly greater in the ΔKPQ2 cell line.

### Propranolol shifts SSI of mutant channels in the hyperpolarizing direction

**Figure 3.**
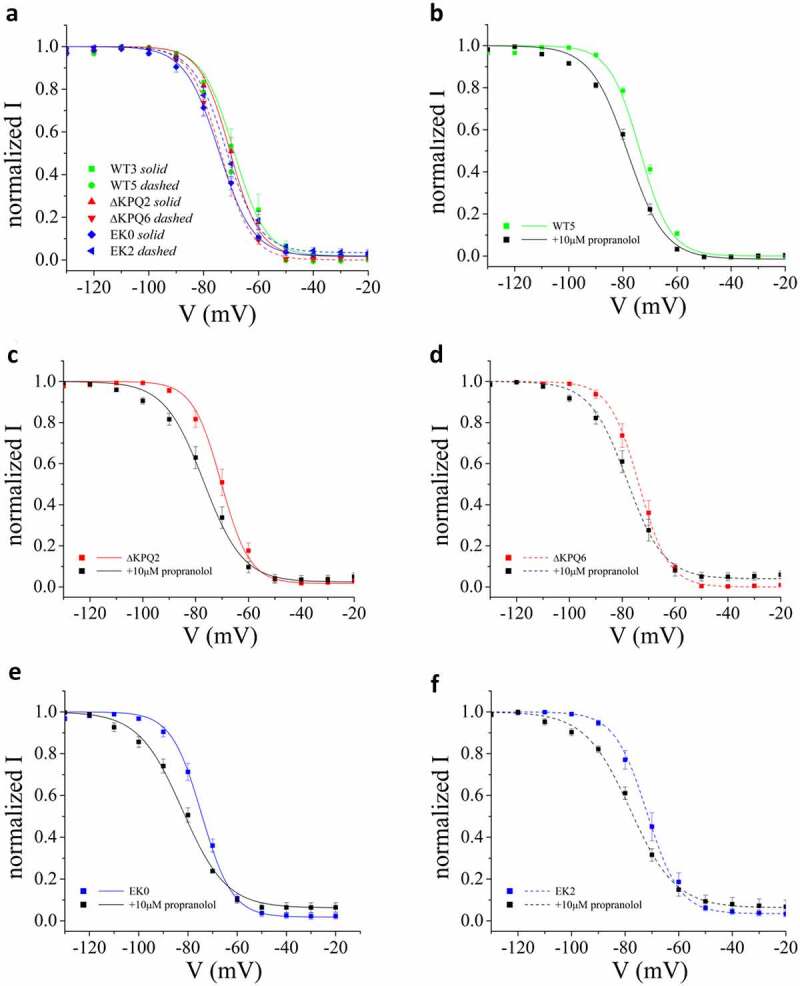
Propranolol causes a negative shift in SSI. A. SSI measured in all WT, ΔKPQ, E1784K cell lines as indicated in the figure. B-D. The influence of propranolol (10 μM) on SSI for all cell lines studied.

The voltage-dependence of steady state inactivation will impact the effects of propranolol on I_NaP_ as demonstrated in previous experiments in which Na^+^ channels were studied using heterologous expression [36]. Since we found I_NaP_ to be more sensitive to propranolol inhibition in cells expressing ΔKPQ vs. E1784K Na^+^ channels vs. cells expressing WT channels, we next investigated the voltage dependence of steady state Na^+^ channel inactivation (SSI) and the effects of propranolol on SSI in each cell line studied. Interestingly, we found on average, a negative shift of about −5 mV of SSI measured in E1784K expressing cells but no shift in SSI ΔKPQ expressing cells (Figure 3(a)). The effects of these mutations on the Voltage-dependence of Na_V_1.5 channel SSI in iPSC myocytes is less than the effects on SSI reported in heterologous expression systems [25–27,36]. As such, we replicated the observed SSI in heterologous expression to understand if it was due to changes in solutions in our study or something endogenous in iPSC myocytes (Supplemental Figure S1). These results suggest that this effect was not due to our experimental solutions. The propranolol-induced shift in SSI for WT, ΔKPQ, and E1784K cells is illustrated in Figure 3 panels B through F. This change in the voltage-dependence of SSI contributes to propranolol inhibition of I_NaP_.

### Propranolol inhibition of IKr is less potent than inhibition of INaL

**Figure 4.**
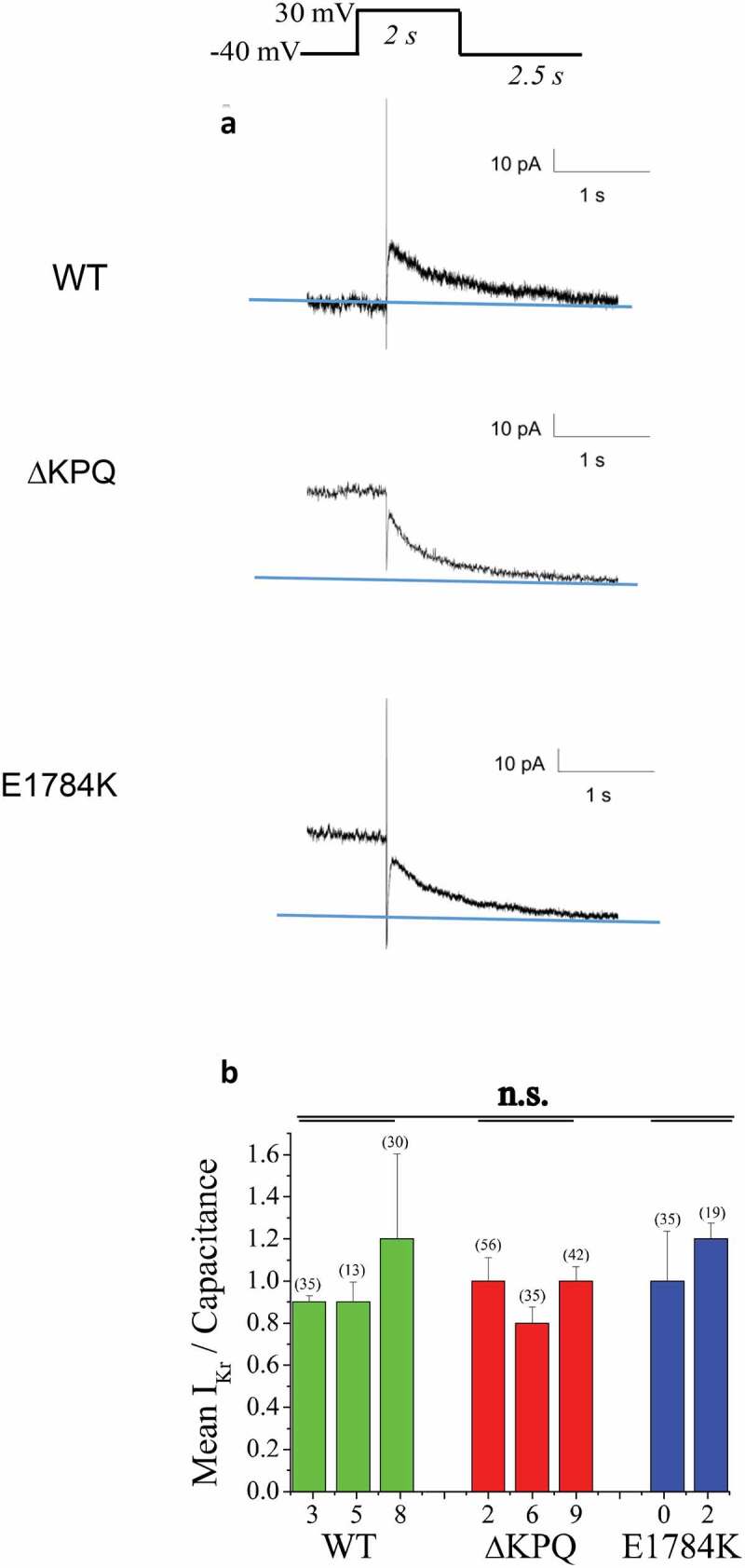
Expression of I_Kr_ in WT and LQT3 patient derived ΔKPQ and E1784K IPSC-CMS. A. Representative I_Kr_ traces in WT, ΔKPQ, and E1784K IPSC-CM. I_Kr_ was determined as E4031-sensitive current in all experiments. The holding potentials were −40 mV. Currents were elicited by pulsing from this holding potential to 30 mV for 2 s. E4031 sensitive peak tail currents following return to the holding potential after the 30 mV pulses were used for measurements and quantification. B. Bar graph summary of I_Kr_ density measured in the number of cells indicated in the figure. There is no significant difference in I_Kr_ density among the groups when cell total populations are grouped into WT, ΔKPQ, and E1784K.

It is well established that off target block of I_Kr_ channels by multiple drugs underlies at least part of drug-induced LQT [37,38] and thus we investigated the effects of propranolol on I_Kr_ in iPSCs expressing E1784K and ΔKPQ channels. Figure 4(a) illustrates measurement of I_Kr_ in each cell line we studied and shows representative I_Kr_ current tail traces for each (WT, ΔKPQ, and E1784K cells from top-to-bottom). Figure 4(b) shows the summary data for peak (initial) I_Kr_ tail amplitude measured at −40 mV following 2 second activation pulses to +10 mV for each line studied. I_Kr_ expression was evident in all lines studied. Expression of this key potassium channel in iPSC myocytes is a very valuable characteristic of these cells because it allows testing for off target effects on these channels in the same cells in which drug modulation of I_NaL_ is investigated.

**Figure 5.**
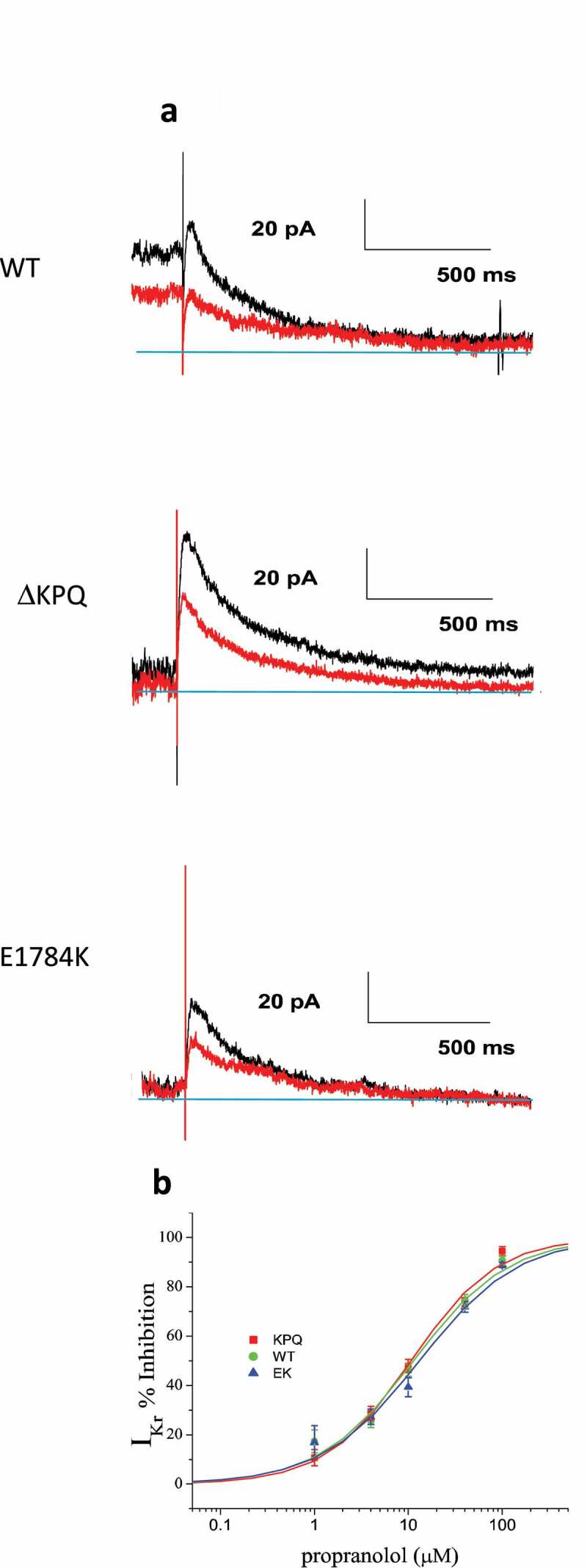
Propranolol inhibits I_Kr_ in WT and LQT3 patient derived ΔKPQ and E1784K IPSC-CM cells. A. Representative I_Kr_ traces recorded as E4031-sensitive current before (black traces) and after application of 10 μM propranolol (red traces) recorded in WT, ΔKPQ, and E1784K IPSC-CMS. B. Dose response curves for total patient derived IPSC-CM populations from 2 different WT donors (WT3, WT8), 3 different LQT3 patients harboring the ΔKPQ deletion (ΔKPQ2, ΔKPQ6, and ΔKPQ9) and 2 different E1784K mutation carrying LQT3 patients (EK0 and EK2). Propranolol IC_50_ for I_Kr_ inhibition in combined cell populations: WT IPSC-CM = 11.35 μM; ΔKPQ IPSC-CMs = 10.68 μM; E1784K IPSC-CMs = 13.12 μM.

We next investigated the effects of propranolol on I_Kr_ expression and have summarized our findings in Figure 5. Figure 5(a) illustrates representative I_Kr_ tail traces recorded in the absence (black traces) and presence of 10 μM propranolol (red traces) for cells expressing WT, ΔKPQ, and E1784K channels. Inhibition by propranolol was measured as a function of reduced E4031 sensitive peak tail current as a function of propranolol concentration. Figure 5(b) illustrates average I_Kr_ inhibition as a function of propranolol concentration for each cell type studied. The average data were then fitted with the Hill equation yielding average IC_50_ values for each cell type. Propranolol inhibition IC_50_ values for I_Kr_ in cells expressing WT and mutant Na_V_1.5 channels ranged from 6.98 μM to 17.39 μM (Table 2). However, when recordings were pooled and averaged across cell lines the IC_50_ values extracted from the averaged I_Kr_ propranolol inhibition data were 11.35 μM propranolol for cells expressing WT channels, 10.68 μM for cells expressing ΔKPQ channels, and 13.12 μM for cells expressing E1784K channels (Figure 5(b)).

**Table 2.** I_Kr_ IC_50_ values for patient-derived IPSC-CM populations from two different WT donors (WT3, WT8), three different LQT3 patients harboring the ΔKPQ deletion (ΔKPQ2, ΔKPQ6, and ΔKPQ9), and two different E1784K mutation carrying LQT3 patients (EK0 and EK2).

patient	I_Kr_ propranolol IC_50_ (μM)
WT3	12.65
WT8	10.35
ΔKPQ2	6.98
ΔKPQ6	13.65
ΔKPQ9	11.93
EK0	17.39
EK2	8.98

## Discussion

Congenital long-QT syndrome (LQTS) is now recognized as one of the most common inherited arrhythmia syndromes with 17 different genetic subtypes [5,33]. Beta blockers are now the primary therapy for LQTS [3,39]. As the genetics of congenital LQTS developed and it was clear that the risk of cardiac events for LQTS mutation carriers was dependent on the mutated gene and that for LQT3 patients, arrhythmia risk was most pronounced during bradycardia or rest [16,40,41]. Following identification of bradycardia as a trigger in LQT3, this variant was not considered amendable to beta-blocker therapy and early clinical studies showed no clear benefit of beta blockers [5].

Nonetheless, subsequent preclinical and clinical studies continued to test the antiarrhythmic activity of β-blockers in the treatment of LQT3 patients. These studies have focused on two of the most studied LQT-3 mutations: the ΔKPQ mutation and the E1784K mutation. These two Na^+^ channel mutations are the focus of the present study. In experiments using an established mouse model for LQT3 in which the efficacy of propranolol on preventing arrhythmias in ΔKPQ-SCN5A knock-in mice, Calvillo et al. found that β-blockade effectively prevented ventricular arrhythmias in this mouse model [30]. However, the mechanism underlying this antiarrhythmic action via anti-adrenergic effects of propranolol or via propranolol inhibition of I_NaL_ was not investigated in this study [31]. In addition, a major clinical study testing the effectiveness of propranolol on arrhythmias in a large multicenter study of LQT3 patients in which the largest number of mutation carriers were carriers of ΔKPQ mutation carriers (66) and E1784K mutation carriers (70). The major conclusion of this study was that β-blocker therapy reduced the risk of cardiac events in female patients with too few male patients participating with cardiac events [32].

To gain further mechanistic insights into these pre-clinical and clinical investigations of the effects of propranolol on Na^+^ channels with ΔKPQ or E1748K mutations or patients harboring these mutations, we investigated the effects of propranolol on Na^+^ channels expressed in iPSC myocytes derived from patients with these LQT3 mutations. We found that propranolol indeed targets I_NaL_ with half maximal inhibitory concentrations of 3.36 μM (ΔKPQ) and 1.58 μM (E1784K). This supports its usefulness of treating LQT3 patients [14]. Propranolol also causes a negative shift of Na^+^ channel SSI that reduces the availability of Na^+^ channels at diastolic potentials for the generation of impulse conduction via I_NaP_. Interestingly, our measured IC_50_ for propranolol I_NaL_ inhibition in iPSC myocytes agrees well with measurement of its inhibition of I_NaL_ carried by transfected mammalian cells expressing ΔKPQ channels (IC_50_ = 2.4 μM) [36]. Importantly, the half maximal concentrations for I_NaL_ inhibition of I_NaL_ were found to be 3.2 (E1748K) and 8.3 (E1748K) times lower than propranolol inhibition of the inwardly rectifying HERG K^+^ channel current I_Kr_. These results indicate that propranolol can be effective and safe in treating LQT3 patients with these common mutations, based at least in part on its inhibition of I_NaL_ provided concentrations used in treating LQT3 patients are in the range of the IC_50_ values that we have measured for I_NaL_ inhibition. A similar propranaolol concentration range has been shown effective at I_NaL_ inhibition in iPSC myocytes expressing a different LQT3 mutation, *SCN5A*-N1774D [42]. We note that these concentrations seem high compared with multiple reports of clinically used doses of propranolol. For example, in a classic study of the clinical pharmacology of propranolol, concentration ranges of up to 800 nM propranolol were reportedly [43]. However, multiple studies of patients investigated in clinical studies have reported serum concentrations over broad ranges from low concentrations of 0.24 μM [44] and 0.36 μM [45] to moderate and high concentrations of 1.54 μM [46]. In this important context Roden and Colleagues have studied concentration-response effects of propranolol on electrophysiological parameters in human subjects and found antiarrhythmic activity at propranolol concentrations of 474 ng/ml (1.83 μM) which is on the order of the IC_50_ values measured for propranolol I_NaL_ inhibition we report here [47], a finding expanded by Ahrens-Nicklas and Clancy, using computational modeling [48]. Our work thus provides experimental evidence that propranolol inhibits I_NaL_ in this concentration range, and that, in this range of concentrations, I_NaL_ inhibition is not accompanied by significant inhibition of I_Kr_ off target effects.

## Supplementary Material

Supplemental Material

## References

[1] Jervell A, Lange-Nielsen F. Congenital deaf-mutism, functional heart disease with prolongation of the Q-T interval and sudden death. Am Heart J. 1957;54(1):59–68. 13435203 10.1016/0002-8703(57)90079-0

[2] Schwartz PJ, Spazzolini C, Crotti L, et al. The Jervell and Lange-Nielsen syndrome: natural history, molecular basis, and clinical outcome. Circulation. 2006;113(6):783–790. 16461811 10.1161/CIRCULATIONAHA.105.592899

[3] Ackerman MJ, Priori SG, Dubin AM, et al. Beta-blocker therapy for long QT syndrome and catecholaminergic polymorphic ventricular tachycardia: are all beta-blockers equivalent? Heart Rhythm. 2017;14(1):e41–e44. 27659101 10.1016/j.hrthm.2016.09.012

[4] Schwartz PJ, Ackerman MJ, Wilde AAM. Channelopathies as Causes of Sudden Cardiac Death. Card Electrophysiol Clin. 2017;9(4):537–549. 29173400 10.1016/j.ccep.2017.07.005

[5] Wilde AAM, Amin AS. Clinical spectrum of SCN5A mutations: long QT syndrome, Brugada syndrome, and cardiomyopathy. JACC Clin Electrophysiol. 2018;4(5):569–579. 29798782 10.1016/j.jacep.2018.03.006

[6] Bohnen MS, Peng G, Robey SH, et al. Molecular pathophysiology of congenital long QT syndrome. Physiol Rev. 2017;97(1):89–134. 27807201 10.1152/physrev.00008.2016 PMC5539372

[7] Keating M. Linkage analysis and long QT syndrome. Using genetics to study cardiovascular disease. [Review]. Circulation. 1992;85(6):1973–1986. J1 - Circ. 1350520 10.1161/01.cir.85.6.1973

[8] Wang Q, Curran ME, Splawski I, et al. Positional cloning of a novel potassium channel gene: KVLQT1 mutations cause cardiac arrhythmias. Nat Genet. 1996;12(1):17–23. 8528244 10.1038/ng0196-17

[9] Jiang C, Atkinson D, Towbin JA, et al. Two long QT syndrome loci map to chromosomes 3 and 7 with evidence for further heterogeneity [see comments]. Nat Genet. 1994;8(2):141–147. 7842012 10.1038/ng1094-141

[10] Curran ME, Splawski I, Timothy KW, et al. A molecular basis for cardiac arrhythmia: HERG mutations cause long QT syndrome. Cell. 1995;80(5):795–803. 7889573 10.1016/0092-8674(95)90358-5

[11] Kannankeril P, Roden DM, Darbar D. Drug-induced long QT syndrome. Pharmacol Rev. 2010;62(4):760–781. 21079043 10.1124/pr.110.003723 PMC2993258

[12] Sanguinetti MC, Tristani-Firouzi M. hERG potassium channels and cardiac arrhythmia. Nature. 2006;440(7083):463–469. 16554806 10.1038/nature04710

[13] Wang Q, et al. SCN5A mutations associated with an inherited cardiac arrhythmia, long QT syndrome. Cell. 1995;80(5):805–811. 7889574 10.1016/0092-8674(95)90359-3

[14] Moreno JD, Clancy CE. Pathophysiology of the cardiac late Na current and its potential as a drug target. J Mol Cell Cardiol. 2012;52(3):608–619. 22198344 10.1016/j.yjmcc.2011.12.003 PMC3816394

[15] Makielski JC. Late sodium current: a mechanism for angina, heart failure, and arrhythmia. Trends Cardiovasc Med. 2016;26(2):115–122. 26092781 10.1016/j.tcm.2015.05.006 PMC5603905

[16] Schwartz PJ, Priori SG, Spazzolini C, et al. Genotype-phenotype correlation in the long-QT syndrome: gene-specific triggers for life-threatening arrhythmias. Circulation. 2001;103(1):89–95. 11136691 10.1161/01.cir.103.1.89

[17] Priori SG, Blomström-Lundqvist C, Mazzanti A, et al. 2015 ESC guidelines for the management of patients with ventricular arrhythmias and the prevention of sudden cardiac death: the task force for the management of patients with ventricular arrhythmias and the prevention of sudden cardiac death of the European Society of Cardiology (ESC). Endorsed by: Association for European Paediatric and Congenital Cardiology (AEPC). Eur Heart J. 2015;36(41):2793–2867. 26320108 10.1093/eurheartj/ehv316

[18] Moss AJ, Kass RS. Long QT syndrome: from channels to cardiac arrhythmias. J Clin Invest. 2005;115(8):2018–2024. 16075042 10.1172/JCI25537 PMC1180552

[19] Papa A, Kushner J, Marx SO. Adrenergic regulation of calcium channels in the heart. Annu Rev Physiol. 2022;84(1):285–306. 34752709 10.1146/annurev-physiol-060121-041653 PMC9573788

[20] Barsheshet A, Goldenberg I, O-Uchi J, et al. Mutations in cytoplasmic loops of the KCNQ1 channel and the risk of life-threatening events: implications for mutation-specific response to beta-blocker therapy in type 1 long-QT syndrome. Circulation. 2012;125(16):1988–1996. 22456477 10.1161/CIRCULATIONAHA.111.048041 PMC3690492

[21] Goldenberg I, Thottathil P, Lopes CM, et al. Trigger-specific ion-channel mechanisms, risk factors, and response to therapy in type 1 long QT syndrome. Heart Rhythm. 2012;9(1):49–56. 21871251 10.1016/j.hrthm.2011.08.020

[22] Mullally J, Goldenberg I, Moss AJ, et al. Risk of life-threatening cardiac events among patients with long QT syndrome and multiple mutations. Heart Rhythm. 2013;10(3):378–382. 23174487 10.1016/j.hrthm.2012.11.006 PMC3690288

[23] Priori SG, et al. Association of long QT syndrome loci and cardiac events among patients treated with beta-blockers. JAMA. 2004;292(11):1341–1344. 15367556 10.1001/jama.292.11.1341

[24] Moss AJ, Zareba W, Hall WJ, et al. Effectiveness and limitations of beta-blocker therapy in congenital long-QT syndrome. Circulation. 2000;101(6):616–623. 10673253 10.1161/01.cir.101.6.616

[25] Wei J, Wang DW, Alings M, et al. Congenital long-QT syndrome caused by a novel mutation in a conserved acidic domain of the cardiac Na + channel. Circulation. 1999;99(24):3165–3171. 10377081 10.1161/01.cir.99.24.3165

[26] Deschenes I, and Baroudi G, Berthet M, et al. Electrophysiological characterization of SCN5A mutations causing long QT (E1784K) and Brugada (R1512W and R1432G) syndromes. Cardiovasc Res. 2000;46(1):55–65. 10727653 10.1016/s0008-6363(00)00006-7

[27] Makita N, Behr E, Shimizu W, et al. The E1784K mutation in SCN5A is associated with mixed clinical phenotype of type 3 long QT syndrome. J Clin Invest. 2008;118(6):2219–2229. 18451998 10.1172/JCI34057 PMC2350431

[28] Bennett PB, Yazawa K, Makita N, et al. Molecular mechanism for an inherited cardiac arrhythmia. Nature. 1995;376(6542):683–685. 7651517 10.1038/376683a0

[29] An RH, Bangalore R, Rosero SZ, et al. Lidocaine block of LQT-3 mutant human Na + channels. Circ Res. 1996;79(1):103–108. 8925557 10.1161/01.res.79.1.103

[30] Calvillo L, Spazzolini C, Vullo E, et al. Propranolol prevents life-threatening arrhythmias in LQT3 transgenic mice: implications for the clinical management of LQT3 patients. Heart Rhythm. 2014;11(1):126–132. 24135497 10.1016/j.hrthm.2013.10.029 PMC3882517

[31] Fabritz L, Franz MR, Carmeliet E, et al. To the Editor–Propranolol, a beta-adrenoreceptor blocker, prevents arrhythmias also by its sodium channel blocking effect. Heart Rhythm. 2014;11(3):e1. 10.1016/j.hrthm.2013.12.027 24361342

[32] Wilde AA, Moss AJ, Kaufman ES, et al. Clinical aspects of type 3 long-QT syndrome: an International multicenter study. Circulation. 2016;134(12):872–882. 27566755 10.1161/CIRCULATIONAHA.116.021823 PMC5030177

[33] Ahn J, Kim HJ, Choi J-I, et al. Effectiveness of beta-blockers depending on the genotype of congenital long-QT syndrome: a meta-analysis. PLoS One. 2017;12(10):e0185680. 29059199 10.1371/journal.pone.0185680 PMC5653191

[34] Terrenoire C, Wang K, Chan Tung KW, et al. Induced pluripotent stem cells used to reveal drug actions in a long QT syndrome family with complex genetics. J Gen Physiol. 2013;141(1):61–72. 23277474 10.1085/jgp.201210899 PMC3536519

[35] Yazawa M, Hsueh B, Jia X, et al. Using induced pluripotent stem cells to investigate cardiac phenotypes in Timothy syndrome. Nature. 2011;471(7337):230–234. 21307850 10.1038/nature09855 PMC3077925

[36] Bankston JR, Kass RS. Molecular determinants of local anesthetic action of beta-blocking drugs: implications for therapeutic management of long QT syndrome variant 3. J Mol Cell Cardiol. 2010;48(1):246–253. 19481549 10.1016/j.yjmcc.2009.05.012 PMC2813422

[37] Roden DM, Wood AJJ. Drug-induced prolongation of the QT interval. N Engl J Med. 2004;350(10):1013–1022. 14999113 10.1056/NEJMra032426

[38] Sanguinetti MC, Mitcheson JS. Predicting drug-hERG channel interactions that cause acquired long QT syndrome. Trends Pharmacol Sci. 2005;26(3):119–124. 15749156 10.1016/j.tips.2005.01.003

[39] Mizusawa Y, Horie M, Wilde AA. Genetic and clinical advances in congenital long QT syndrome. Circ J. 2014;78(12):2827–2833. 25274057 10.1253/circj.cj-14-0905

[40] Clancy CE, Tateyama M, Kass RS. Insights into the molecular mechanisms of bradycardia-triggered arrhythmias in long QT-3 syndrome. J Clin Invest. 2002;110(9):1251–1262. 12417563 10.1172/JCI15928 PMC151612

[41] Schwartz PJ. Management of long QT syndrome. Nat Clin Pract Cardiovasc Med. 2005;2(7):346–351. 16265560 10.1038/ncpcardio0239

[42] Hirose S, Makiyama T, Melgari D, et al. Propranolol attenuates late sodium current in a long QT syndrome type 3-human induced pluripotent stem cell model. Front Cell Dev Biol. 2020;8:761. 32903469 10.3389/fcell.2020.00761 PMC7438478

[43] Nies AS, Shand DG. Clinical pharmacology of propranolol. Circulation. 1975;52(1):6–15. 1093761 10.1161/01.cir.52.1.6

[44] Salehifar E, Ebrahim S, Shiran M-R, et al. Pharmacokinetic parameters and over-responsiveness of Iranian population to propranolol. Adv Pharm Bull. 2017;7(2):195–202. 28761821 10.15171/apb.2017.024 PMC5527233

[45] Wojcicki J, Jaroszynska M, Droździk M, et al. Comparative pharmacokinetics and pharmacodynamics of propranolol and atenolol in normolipaemic and hyperlipidaemic obese subjects. Biopharm Drug Dispos. 2003;24(5):211–218. 12784321 10.1002/bdd.357

[46] Cales P, Grasset D, Ravaud A, et al. Pharmacodynamic and pharmacokinetic study of propranolol in patients with cirrhosis and portal hypertension. Br J Clin Pharmacol. 1989;27(6):763–770. 2569324 10.1111/j.1365-2125.1989.tb03438.x PMC1379803

[47] Duff HJ, Roden DM, Brorson L, et al. Electrophysiologic actions of high plasma concentrations of propranolol in human subjects. J Am Coll Cardiol. 1983;2(6):1134–1140. 6630784 10.1016/s0735-1097(83)80340-4

[48] Ahrens-Nicklas RC, Clancy CE, and Christini DJ. Re-evaluating the efficacy of {beta}-adrenergic agonists and antagonists in long QT-3 syndrome through computational modelling. In Cardiovasc Res. 2009. Vol. 82(3). p. 439-47. 10.1093/cvr/cvp083 PMC2682617 19264765

