# Supplementary material for: Exploring mutation specific beta blocker pharmacology of the pathogenic late sodium channel current from patient-specific pluripotent stem cell myocytes derived from long QT syndrome mutation carriers
Source: Channels (Austin). 2022 Aug 10;16(1):173–84. doi: 10.1080/19336950.2022.2106025 (PMC9373745; doi:10.1080/19336950.2022.2106025)
Supplement: Supplemental Material [file KCHL_A_2106025_SM7886.docx]

Supplemental Figure S1.


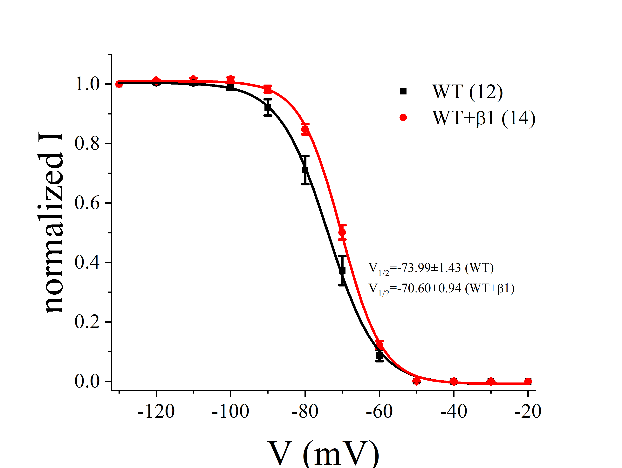

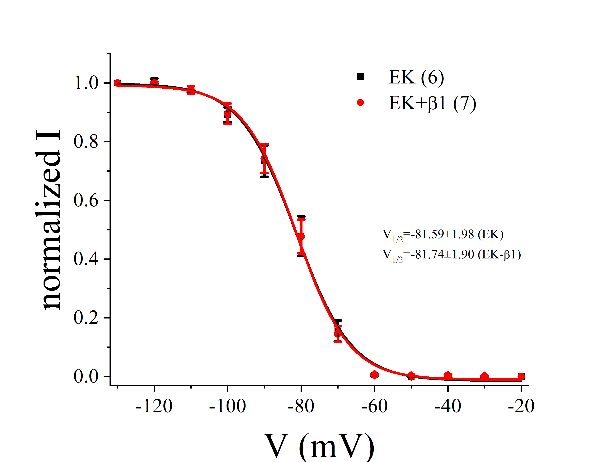

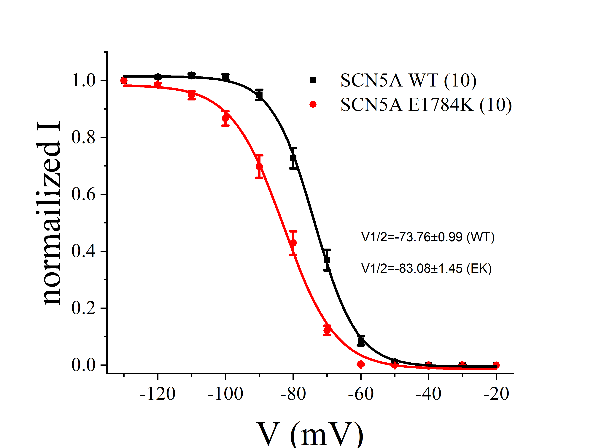


**Supplemental Figure S1**. Impact of co-expression of alpha and beta subunits on I_NaP_ Steady State Inactivation (SSI). Left: Comparison of wild type Na_V_1.5 SSI with and without β1 subunit expression in HEK293 cells; Middle: Comparison of E1784K Na_V_1.5 SSI with and without β1 subunit expression in HEK293 cells; right: Comparison of SSI in wild type vs E1784K Na1.5 in HEK293 cells.
